# Individuals living in an onchocerciasis focus and treated three-monthly with ivermectin develop fewer new onchocercal nodules than individuals treated annually

**DOI:** 10.1186/s13071-020-04126-x

**Published:** 2020-05-15

**Authors:** Jérémy T. Campillo, Cédric B. Chesnais, Sébastien D. S. Pion, Jacques Gardon, Joseph Kamgno, Michel Boussinesq

**Affiliations:** 1 UMI 233, Institut de Recherche pour le Développement (IRD) and University of Montpellier 1, 911 avenue Agropolis, P.O. Box 64501, 34394 Montpellier Cedex 5, France; 2 Hydrosciences Montpellier, Institut de Recherche pour le Développement (IRD), Montpellier, France; 3 Centre for Research on Filariasis and other Tropical Diseases (CRFilMT), P.O. Box 5797, Yaoundé, Cameroon; 4 Faculty of Medicine and Biomedical Sciences, University of Yaoundé 1, Yaoundé, Cameroon

**Keywords:** Onchocerciasis, *Onchocerca volvulus*, Nodules, Onchocercoma, Ivermectin, Cameroon

## Abstract

**Background:**

Little information is available on the effect of ivermectin on the third- and fourth-stage larvae of *Onchocerca volvulus*. To assess a possible prophylactic effect of ivermectin on this parasite, we compared the effects of different ivermectin regimens on the acquisition of onchocercal nodules.

**Methods:**

We analyzed data from a controlled randomized clinical trial of ivermectin conducted in the Mbam Valley (Cameroon) between 1994 and 1998 in a cohort of onchocerciasis infected individuals. The number of nodules that appeared between the start and the end of the clinical trial was analyzed, using ANOVA and multivariable Poisson regressions, between four treatment arms: 150 µg/kg annually, 800 µg/kg annually, 150 µg/kg 3-monthly, and 800 µg/kg 3-monthly.

**Results:**

The mean number of nodules that appeared during the trial was reduced by 17.7% in subjects treated 3-monthly compared to those treated annually (regardless of the dose). Poisson regression model, adjusting on subject’s age and weight, initial number of nodules and intensity of *O. volvulus* infection in his village of residence, confirmed that the incidence of new nodules was reduced in 3-monthly treatment arms compared to annually treatment arms, and that the dosage of ivermectin does not seem to influence this effect. Furthermore, the number of newly acquired nodules was positively associated with the initial number of nodules. Analysis of disappearance of nodules did not show any significant difference between the treatment groups.

**Conclusions:**

To our knowledge, these results suggest for the first time in humans, that ivermectin has a partial prophylactic effect on *O. volvulus*. Three-monthly treatment seems more effective than annual treatment to prevent the appearance of nodules.
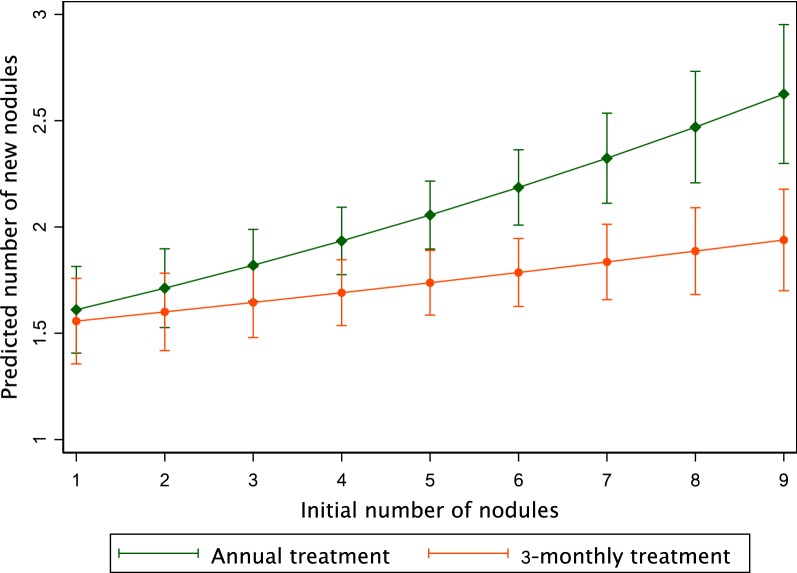

## Background

Human onchocerciasis, also called “river blindness”, is a neglected tropical disease (NTD) caused by the filarial nematode *Onchocerca volvulus*. In 1995, the World Health Organization (WHO) launched the African Programme for Onchocerciasis Control (APOC), which was mainly based on mass drug administration (MDA) of ivermectin (IVM). IVM, which is usually given annually at the standard dose of 150 µg/kg of body weight, has a direct effect on the microfilariae (mf) present in the skin (microfilaricidal effect) and prevents the release of new mf by the adult female worms for several months (embryostatic effect). However, the effect of IVM on the viability of the adult worms (macrofilaricidal effect) is considered as moderate and treatments have to be repeated every year (or at shorter intervals) to maintain skin microfilarial densities (MFD) at low levels not associated with clinical manifestations. Besides this, little information is available on the effect of IVM on the third- and fourth-stage larvae (L3s and L4s) which develop to the adult stage during the first months following the bite of an infective blackfly, and on the immature adults. The effect on these L3s, L4s, and immature adults, which would prevent the development up to the stage of fecund adult worms releasing mf, has been called causal prophylaxis, or suppressive effect [1]. We will use the term “prophylactic effect” throughout the text below.

Only four *in vivo* studies were conducted to evaluate the prophylactic effect of IVM on *Onchocerca* spp. The first one included 18 chimpanzees experimentally infected with *O. volvulus*. Six animals were treated with IVM (at 200 µg/kg) on the day of inoculation of the L3s, six were treated on day 28 and six were not treated. After having followed-up the development of infection by repeated skin biopsies for 30 months, the authors concluded that IVM could have a partial effect on the L3s (which live for about two to three days in the definitive host before molting to the L4 stage [2]), but no effect on the L4s [3]. The second study was conducted in an area of North Cameroon where the cattle parasite *O. ochengi* is endemic. Two groups of calves between two and eight weeks of age were treated monthly with subcutaneous IVM (Ivomec®) at either 200 µg/kg or 500 µg/kg for 21 months, and a third group was left untreated. Before each treatment, the animals were palpated for *O. ochengi* nodules and underwent a skin biopsy. The fact that none of the 15 treated calves developed adult worm infection, whereas five of the six control calves became infected led the authors to conclude that IVM had an effect on the L3s and L4s of *O. ochengi* [4]. The third study was conducted in an onchocerciasis hyperendemic focus (Mbam valley, Cameroon) and included human subjects with no *O. volvulus* mf in skin biopsies (“skin snips” taken with a 2 mm Holth punch). These patients were treated, just after the start of the high transmission period, with either a single oral dose of IVM (150 µg/kg) plus ferrous sulphate tablets, or the latter drug only. One year after, the incidence of *O. volvulus* microfilaridermia was 23.4% in the IVM group and 25.8% in the control group, and the mean MFD were similar in the two groups (2.2 and 2.7 mf per skin snip, respectively). The authors concluded that a single dose of IVM had no perceptible prophylactic effect in this highly endemic area [5]. The fourth study included calves (mean age: 9 weeks) naturally exposed to *O*. *ochengi* infection, and treated with IVM at monthly or 3-monthly intervals, or left untreated. After 22 months of exposure, 11 of the 14 control animals had acquired nodules (including ten with skin mf), two of the ten animals treated 3-monthly had nodules (but no skin mf), and none of the ten animals treated at monthly interval had acquired nodules [6]. These results suggest that 3-monthly treatment has a partial prophylactic effect on *O. ochengi*. Besides these trials, the effect of IVM on L3s and the L3-L4 molting process was supported by *in vitro* studies using *Onchocerca lienalis* [7, 8].

A double-blind randomized controlled trial aimed at assessing the potential macrofilaricidal effect of high (400–800 µg/kg) and/or more frequent (3-monthly) doses of IVM on *O. volvulus* was conducted in the Mbam valley (Cameroon) between 1994 and 1998. This effect was evaluated by the histologic examination of sections of nodules collected at the outset and at the end of the trial [9]. The proportion of dead female worms was found to be higher in the nodules collected from subjects treated 3-monthly than in those treated annually. During this trial, a careful examination for all palpable nodules was conducted at the outset of the trial, and during the nodulectomy round organized in 1997. The number and the location of each palpated nodule was noted on a standard chart. In the present paper, we present the results of statistical analyses performed on the number of nodules which had appeared or disappeared between the two examination rounds. Our main objective was to assess whether high doses or more frequent IVM treatment was associated with a lower number of new nodules (suggesting a prophylactic effect). Analyses were also performed on the number of nodules that had spontaneously disappeared.

## Methods

### Study population and subjects

The protocol of the trial has been described in detail elsewhere [9]. Briefly, it was conducted in the Bafia health district, located in the onchocerciasis hyperendemic focus of the Mbam Valley (Cameroon). Eligible subjects were males aged 18–60 years-old in a good state of health, with no contra-indication to IVM, and who presented at least two palpable nodules at the outset of the trial.

### Procedures

After having signed an informed consent form, subjects were randomly allocated to one of the four treatment groups receiving either 150 µg/kg annually (control group), or high dose (400 µg, then 800 µg/kg) annually, or 150 µg/kg 3-monthly or high dose 3-monthly. The pre-treatment nodulectomy round was performed in May-June 1994. Before the nodulectomy, each participant was carefully examined and the location of each palpable nodule was noted on a standard anatomic chart. The randomly selected nodule to remove during the operation was represented by a green dot on the chart, and all the others were noted as a red dot. After nodulectomy, each participant received a 150 µg/kg “clearing dose” of IVM to avoid the possibility of severe reactions developing in any patients subsequently taking their first dose on the high-dose regimen. The 3-year courses of treatment under investigation began in August 1994, 2–3 months after the clearing dose. A total of 643 subjects participated in this treatment round. The second round of nodulectomy was organized in August 1997. A total of 102 subjects was lost between August 1994 and August 1997 (24 deaths, 17 excluded on medical grounds and 61 subjects who moved away or were excluded because they missed one treatment round). The number of subjects participating in the second nodulectomy round was thus 541. Before the collection of the nodules, each patient was re-examined and the location of the nodules present was noted on the anatomic chart used in 1994. The nodules which had spontaneously disappeared in the interval were noted, and the location of the nodules which had appeared between 1994 and 1997 (thereafter called “new nodules”) were noted by a blue dot. As these examinations had been performed just before the operation, an additional clinical examination was performed in November 1997, i.e. in a less time-constrained context, to confirm the results obtained three months before. This examination could be performed in 485 subjects and the statistical analyses were conducted on the latter.

### Statistical analysis

**Fig. 1:**
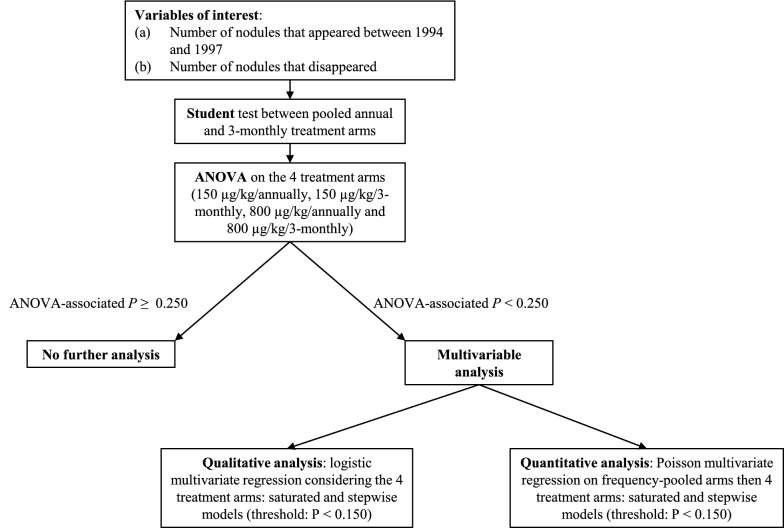
Statistical analysis plan

Variables of interest were defined as (i) the number of nodules which appeared between 1994 and 1997 and (ii) the number of nodules which disappeared. For these two variables, we used the same statistical analysis plan (Fig. 1). First, we assessed the difference between people treated annually and people treated 3-monthly, regardless of the dose, using Studentʼs t-test. Then, comparisons were performed between the four treatment arms using an ANOVA; Bonferroni test was subsequently used to assess which treatment arm(s) differed from the others. In case of an ANOVA-associated *P*-value < 0.250, we performed a multivariable analysis to assess the possible associations between appearance of new nodules and the following variables: treatment group as a categorical variable (first considering annual arms *versus* 3-monthly arms, then considering the four treatment arms), subject’s age (continuous variable), subject’s weight (continuous variable), initial number of palpable nodules (expressed sequentially as a continuous variable and as a categorical variable using the following categories: < 5 nodules and ≥ 5 nodules), subject’s initial microfilarial density (MFD, expressed as the number of mf per milligram of skin), and the intensity of *O. volvulus* infection in the participant’s village of residence (proxy of exposure to infection). The individual MFD was calculated as the ratio of the sum of the number of mf found in both snips (taken from the left and right iliac crests) to the sum of the weights of each snip. In the analyses, the MFD was expressed as a categorical variable using the interquartile categories: < 30, 31–80, 81–170, and > 170 mf/mg. The intensity of *O. volvulus* infection in the participant’s village of residence was expressed in three categories (low, moderate and high intensity) and was defined according to the community microfilarial load (CMFL) in the community measured during previous parasitological surveys in the community itself, or in neighbouring communities located at the same distance from the Mbam River. The CMFL, a classical indicator used to express the intensity of infection with *O. volvulus* in a community, corresponds to the Williams’ geometric mean of the individual MFDs (here expressed as number of mf per skin snip) in all subjects examined (not only those skin snip positive) aged ≥ 20 years-old [10]. The three categories used in the analyses correspond to CMFL < 20 mf/skin snip, 20–50 mf/skin snip and ≥ 50 mf/skin snip. Multivariable logistic regression models were used with the dependent variable coded as a binary outcome (appearance of nodules *versus* no appearance of nodule). The reference group is defined as people with less than 5 nodules in the 150 µg/kg 3-monthly treatment group. Then, Poisson regression models were used, with the dependent variable recoded as 0, 1, 2, 3, 4 and ≥ 5 new nodules. We constructed two models: one with the 4 treatment arms and one with the pooled annual and 3-monthly treatment arms, regardless of the dose. For all models, an interaction term between the treatment arm and the initial number of nodules was added to assess the mean number of new nodules according to these two variables simultaneously. For each regression model, we presented two procedures: a saturated model with all explicative variables, whatever their *P*-values and a backward stepwise procedure with a *P*-value threshold of 0.150 for the explicative variables. We presented regression coefficients and their 95% confidence intervals (95% CI). The reference group is defined as people who had the lowest number of initial nodules (i.e. 2 nodules) in the 150 µg/kg annually group or in the annual treatment, depending on the model. According to this regression analysis, predictions were made using the *margins* and *marginsplot* commands.

All analyses were performed using the STATA v.15.1 software (StatCorps, LP, College Station, TX, USA).

## Results

### Baseline characteristics

Table 1 presents the baseline characteristics of the 485 study subjects as a whole and in each treatment arm. Before the first nodulectomy round, the participants of the four treatment groups were similar in terms of age (Kruskal–Wallis H-test: *χ*^2^ = 1.36, *df* = 3, *P* = 0.714), body weight (Kruskal–Wallis H-test: *χ*^2^ = 4.98*, df* = 3, *P* = 0.172), mean number of nodules (Kruskal–Wallis H-test: *χ*^2^ = 3.49, *df* = 3, *P* = 0.349), CMFL in the village of residence (Chi-square test: *χ*^2^ = 4.77, *df* = 6, *P* = 0.574) and MFD (Kruskal–Wallis H-test: *χ*^2^ = 2.39, *df* = 3, *P* = 0.495).

**Table 1:** Baseline characteristics of the subjects included in 1994, before the start of the clinical trial

	All participants	150 µg/kg annually	800 µg/kg annually	150 µg/kg 3-monthly	800 µg/kg 3-monthly
No. of subjects	485	126	122	125	112
Mean age ± SD	36.9 ± 12.0	36.4 ± 11.4	38.1 ± 12.4	36.7 ± 11.9	36.6 ± 12.3
Mean weight ± SD (kg)	63.0 ± 8.0	62.7 ± 8.2	64.3 ± 7.1	62.4 ± 8.5	62.7 ± 8.0
Mean no. of nodules in 1994 ± SD	5.7 ± 2.8	5.5 ± 2.5	5.8 ± 2.3	5.6 ± 3.2	5.9 ± 2.9
Median no. of nodules in 1994 (IQR)	5 (4–7)	5 (4–7)	6 (4–7)	5 (3–7)	6 (4–7)
CMFL in the village of residence:					
Low (*n*, %)	50 (10.3)	10 (7.9)	12 (9.8)	18 (14.4)	10 (8.9)
Middle (*n*, %)	135 (27.8)	39 (31.0)	37 (30.3)	30 (24.0)	29 (25.9)
High (*n*, %)	300 (61.9)	77 (61.1)	73 (59.8)	77 (61.6)	73 (65.2)
Geometric mean of MFD	56.0	64.7	50.8	48.7	61.8
Median of MFD (IQR)	79 (27–171)	91 (34–197)	71 (26–157)	74 (24–143)	84 (28–187)

*Abbreviations*: No, number; SD, standard deviation; IQR, interquartile range; *n*, number of subjects; CMFL, community microfilarial load; MFD, individual microfilarial density (mf/mg)

### Analysis of appearance of nodules between 1994 and 1997

The mean number of new nodules in the 237 subjects treated 3-monthly (1.85) was 17.7% lower than in the 248 subjects treated annually (2.25; t-test: *t*_(483)_ = 2.67, *P* = 0.008). The mean numbers of nodules that appeared between 1994 and 1997 in subjects of each treatment group are shown in Table 2. ANOVA indicated a statistically significant difference in the number of new nodules between the treatment groups (ANOVA: *F*_(3, 484)_ = 3.60, *P* = 0.014). Bonferroni correction showed that the number of new nodules was significantly lower in the group which had received 150 µg/kg 3-monthly than in the group treated with 800 µg/kg annually (*P* = 0.008). No significant difference was found between the two groups treated annually (*P* = 1.000), nor between the two groups treated 3-monthly (*P* = 0.871), nor between the two groups treated with 150 µg/kg (annually *vs* 3-monthly) (*P* = 0.260).

**Table 2:** Appearance and disappearance of the nodules after 3 years of treatment

	All participants	150 µg/kg annually	800 µg/kg annually	150 µg/kg 3-monthly	800 µg/kg 3-monthly	*P*-value
No. of subjects	485	126	122	125	112	
Mean no. of new nodules ± SD	2.1 ± 1.7	2.1 ± 1.7	2.4 ± 1.8	1.7 ± 1.5	2.0 ± 1.6	0.014
Mean no. of nodules that disappeared ± SD	0.5 ± 0.8	0.5 ± 0.8	0.5 ± 0.7	0.6 ± 0.8	0.6 ± 0.8	0.279

*Abbreviation*: No, number; SD, standard deviation

According to the logistic regression analysis (Table 3), the probability to develop new nodules did not differ significantly between the treatment groups of subjects with less than 5 palpable nodules. In addition, people with more than 5 nodules and belonging to the groups treated annually with 150 µg/kg and 800 µg/kg had, respectively, 4.1 and 5.5 higher chance to have new nodule(s) than those treated with 150 µg/kg 3-monthly and with less than 5 nodules. The CMFL in the village of residence and the individual MFD were not found to be associated with the probability of appearance of new nodules.

**Table 3:** Logistic regression of the appearance of nodules

Variables	Saturated model		Stepwise model	
	OR(95% CI)	*P*-value	OR (95% CI)	*P*-value
Appearance of nodule(s) (yes/no)				
Less than 5 nodules:				
150 µg/kg 3-monthly (*n* = 66)	Ref.		Ref.	
150 µg/kg annually (*n* = 77)	1.6 (0.6–3.8)	0.32	1.6 (0.7–3.8)	0.28
800 µg/kg 3-monthly (*n* = 54)	2.6 (0.8–7.7)	0.092	2.6 (0.9–7.9)	0.082
800 µg/kg annually (*n* = 58)	0.9 (0.4–2.1)	0.777	0.9 (0.4–2.2)	0.871
More than 5 nodules				
150 µg/kg 3-monthly (*n* = 59)	0.8 (0.3–1.9)	0.619	0.9 (0.4–2.0)	0.736
150 µg/kg annually (*n* = 49)	3.5 (0.9–13.3)	0.066	4.1 (1.1–15.3)	0.034
800 µg/kg 3-monthly (*n* = 58)	1.2 (0.4–2.9)	0.759	1.3 (0.5–3.2)	0.577
800 µg/kg annually (*n* = 64)	5.2 (1.4–19.5)	0.014	5.5 (1.5–20.1)	0.01
Age	1.0 (1.0–1.0)	0.872		
Weight	1.0 (1.0–1.0)	0.661		
CMFL in the village of residence				
Low	Ref.			
Middle	1.2 (0.5–2.9)	0.704		
High	1.3 (0.6–3.0)	0.537		
MFD				
0–30 mf/mg	Ref.			
31–80 mf/mg	1.3 (0.6–2.6)	0.459		
81–170 mf/mg	1.2 (0.6–2.4)	0.624		
> 171 mf/mg	1.7 (0.8–3.7)	0.151		

Abbreviations: OR, odds-ratio; 95% CI, 95% confidence interval; *n*, number of subjects; CMFL, community microfilarial load; MFD, microfilarial density; Ref., reference

**Fig. 2:**
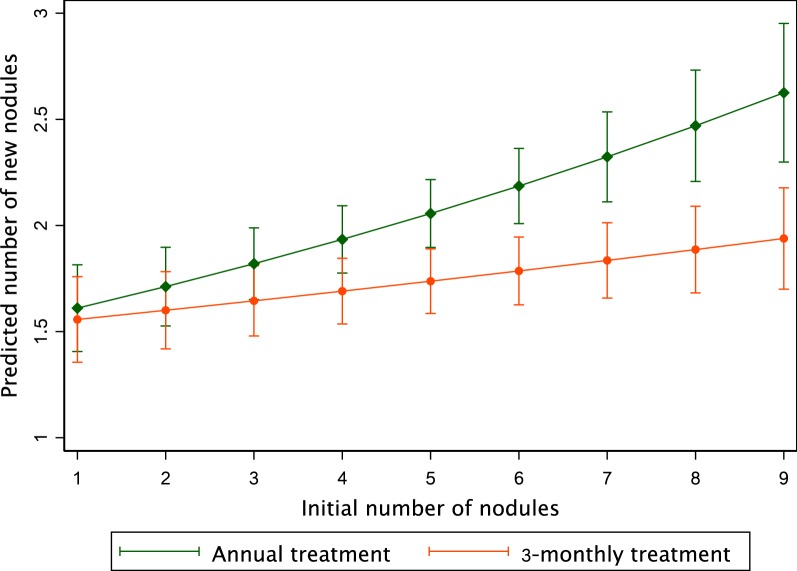
Predicted number of new nodules *vs* initial number of nodules (annual *vs* 3-monthly arms). Bars indicate the 95% confidence interval

**Fig. 3:**
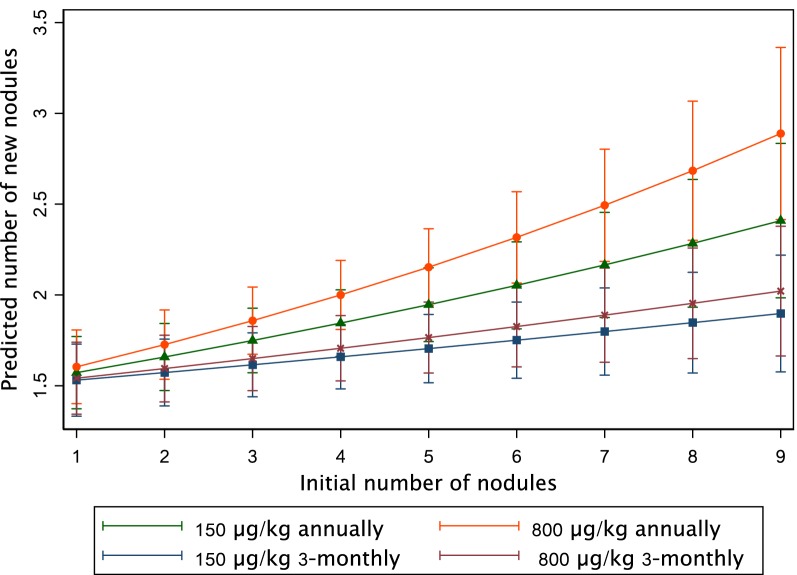
Predicted number of new nodules vs initial number of nodules (four treatment arms). Bars indicate the 95% confidence interval

Table 4 shows the results of the Poisson regression model for each frequency of treatment explaining the count of new nodules. It shows that 3-monthly treatment (regression coefficient = 0.026; 95% CI: 0.005–0.048), whatever the dose, is more than twice as effective as annual treatment (regression coefficient = 0.058; 95% CI: 0.035–0.081) to prevent the appearance of nodules. The difference in the slope is significant (*P* = 0.001). Figure 2 represents the predictions of this model. Table 5 shows the results of the Poisson regression model including the four treatment arms separately. For these two regression models, initial individual MFD was associated with the appearance of new nodules. As shown in Figs. 2 and 3, these models reveal a strong interaction between the initial number of nodules and the predicted number of new nodules. It shows that in subjects treated 3-monthly (either with 150 or 800 µg/kg) fewer nodules had appeared than in subjects treated annually (with 150 or 800 µg/kg), and that the difference of appearance was highly correlated with the initial number of nodules harboured by the participants. In addition, treatment with high IVM dose does not seem to influence the number of new nodules, regardless of the number of initial palpable nodules.

**Table 4:** Poisson regression model with pooled arms

Variable	Saturated model		Stepwise model	
	b (95% CI)	*P-*value	b (95% CI)	*P-*value
Increase in no. of new nodules for each additional initial nodule^a^				
Annual treatment	0.055 (0.030–0.080)	< 0.001	0.058 (0.035–0.081)	< 0.001
3-monthly treatment	0.023 (0.0004–0.046)	0.045	0.026 (0.005–0.048)	0.014
Age	0.005 (0.0001–0.010)	0.045	0.006 (0.002–0.009)	0.002
Weight	− 0.007 (− 0.005–0.003)	0.703		
CMFL in the village of residence				
Low	Ref.			
Middle	0.089 (− 0.153–0.332)	0.470		
High	0.149 (− 0.074–0.372)	0.191		
MFD				
0–30 mf/mg	Ref.		Ref.	
31–80 mf/mg	0.084 (− 0.108–0.277)	0.389	0.102 (− 0.081–0.285)	0.275
81–170 mf/mg	0.265 (0.082–0.447)	0.004	0.290 (0.117–0.463)	0.001
> 171 mf/mg	0.358 (0.181–0.535)	< 0.001	0.383 (0.217–0.550)	< 0.001
*P* of the model	< 0.001		< 0.001	
AIC	1648		1644	
BIC	1686		1669	
Log likelihood	− 815.2		− 816.3	

^a^The reference is defined as annual treatment and 2 initial nodules

*Abbreviations*: b, regression coefficient; 95% CI, 95% confidence interval; Ref., reference; CMFL, community microfilarial load; MFD, microfilarial density; AIC, Akaike’s information criterion; BIC, Bayesian information criterion

**Table 5:** Poisson regression model (with interaction term between initial number of nodules and treatment arms)

Variable	Saturated model		Stepwise model	
	b (95% CI)	*P-*value	b (95% CI)	*P*-value
Increase in no. of new nodules for each additional initial nodule^a^				
150 µg/kg annually	0.043 (0.014–0.071)	0.004	0.046 (0.019–0.073)	0.001
800 µg/kg annually	0.067 (0.039–0.095)	< 0.001	0.070 (0.043–0.096)	< 0.001
150 µg/kg 3-monthly	0.021 (− 0.004–0.047)	0.106	0.024 (− 0.000–0.048)	0.052
800 µg/kg 3-monthly	0.026 (− 0.001–0.053)	0.063	0.029 (0.004–0.056)	0.026
Age	0.005 (− 0.00001–0.010)	0.051	0.006 (0.002–0.009)	0.003
Weight	− 0.001 (− 0.005–0.003)	0.659		
CMFL in the village of residence				
Low	Ref.			
Middle	0.090 (− 0.154–0.333)	0.470		
High	0.151 (− 0.072–0.375)	0.185		
MFD				
0–30 mf/mg	Ref.		Ref.	
31–80 mf/mg	0.089 (− 0.103–0.281)	0.364	0.105 (− 0.079–0.288)	0.263
81–170 mf/mg	0.278 (0.095–0.461)	0.003	0.301 (0.128–0.474)	0.001
> 171 mf/mg	0.371 (0.194–0.549)	< 0.001	0.394 (0.227–0.561)	< 0.001
*P* of the likelihood-ratio test for the interaction term: 0.035				
*P* of the model	< 0.001		< 0.001	
AIC	1649		1645	
BIC	1695		1678	
Log likelihood	− 813.5		− 814.6	

^a^The reference is defined as 150 µg/kg annually and 2 initial nodules

*Abbreviations*: b, regression coefficient; 95% CI, 95% confidence interval; Ref., reference; CMFL, community microfilarial load; MFD, microfilarial density; AIC, Akaike’s information criterion; BIC, Bayesian information criterion

### Analysis of disappearance of nodules between 1994 and 1997

In the 248 subjects treated annually, the mean number of nodules which disappeared between 1994 and 1997 (0.46) was lower than in the 237 subjects treated 3-monthly (0.60) but the difference was not significant (t-test: *t*_(483)_ = − 1.88, *P* = 0.061). The mean numbers of nodules that disappeared between 1994 and 1997 in subjects of each treatment group are shown in Table 2.

The ANOVA did not show any difference between the four groups (ANOVA: *F*_(3, 484)_ = 1.29, *P* = 0.279); consequently, we did not perform further analysis on the disappearance of nodules.

## Discussion

To the best of our knowledge, our study is the first to compare the effect of various IVM treatment regimens on the appearance of new onchocercal nodules in human subjects exposed to transmission of *O. volvulus*. It demonstrates that the mean number of palpable nodules which appeared within the 3-year period of the trial was significantly lower in the individuals treated 3-monthly with IVM than in those treated annually, and that the high doses had no higher effect than standard doses in reducing the number of new nodules. Strangely, the difference between 800 µg/kg annually and 800 µg/kg 3-monthly was less marked than the difference between 150 µg/kg annually and 150 µg/kg 3-monthly, we have no biological clue to understand this difference, it may be due to unmeasured confounding factors (such as pharmacokinetics or pharmacodynamics characteristics) or to hazards due to serendipity.

We found no significant association between CMFL and appearance of nodules, we hypothesise that with a larger sample size, the association between CMFL exposition and appearance of new nodules may appear.

These results should be interpreted in the light of what is known on the biology of *O. volvulus* within the year following the infective bite. The modalities of development of *O. volvulus* from the initial penetration of the parasite into the host as an L3 to the time when it is found as an adult fecund stage in a nodule are not fully known. The time of the L3-L4 molt has been assessed by *in vitro* studies and by infecting experimentally various animals with L3s of *Onchocerca* spp. The time of the final molt was evaluated by following up the appearance of antibodies, which were assumed to be stage-specific, in a simian model, and by an *in vitro* culturing system. It appears that, for *O. volvulus*, the L3-L4 molt starts 2–3 days after inoculation [11], that the final molt occurs between 1.5 and 2.5 months [12], that the adults become sexually mature at 7.5–11 months, and that the first mf produced by the mature adult female worms can be detected after an average period of 12–15 months [13]. Thus, the lifespan of the immature adult worms would range between five months (7.5 minus 2.5) and 9.5 months (11 minus 1.5). Given this timeframe, one can estimate that the proportion of L4s exposed to IVM in an individual treated 3-monthly will be between 50% (if the L4s’ lifespan is 1.5 months) and 83% (if it is 2.5 months). Regarding immature worms, most would be exposed twice, and a small proportion three times, to the drug. Conversely, given their short lifespan, only a small proportion of L3s “inoculated” to these subjects treated every 90 days would be exposed to the drug. If one assumes that the appearance (or not) of new nodules reflects the effect of the drug on the parasitic stages preceding the mature adult stage, which is debatable (see below), the observed decrease of 17.7% in the number of new nodules in the subjects treated 3-monthly suggests that the prophylactic effect of IVM is not limited to the effects of the drug on the L3s or on the L3-L4 molting process only, but that IVM has also a partial effect on the L4s and/or the immature adult worms. IVM is known to kill L4s and young adults of the canine heartworm *Dirofilaria immitis* and to have a “slow kill” activity against *D. immitis* adults. Indeed, IVM is described as a very effective prophylactic drug against *D. immitis* infection in dogs. Furthermore, we know that the latter parasite is a very close relative to *Onchocerca* spp. Thus, we can hypothesise that IVM works in the same way in *O. volvulus* [14].

In this study, we assumed that the effect of a drug on the L4s and immature adults can be assessed by following up the appearance of new nodules in hosts exposed to transmission of *O. volvulus*. This is debatable because the sites where the L4s and the immature adults live, and the modalities by which the adult female worms are finally found in a nodule, are poorly known. In particular, the extent to which immature females are attracted by existing nodules or are able to create a new nodule are not known. According to Duke et al. [11], “it is expected … that the L4 will be highly mobile and capable (by means unknown) of locating the adult sites of election or (perhaps by means of pheromones) of finding pre-existing worm bundles; and that the immature females may continue these wanderings, …, but are then likely to settle down to form nodules of their own or to join pre-existing nodules. The possibility cannot be excluded that some of the immature females may remain dormant at a prepubertal phase, situated in the connective tissues away from the nodules”. Guderian et al. [15] compared the sites of appearance of new nodules in a group of subjects from whom all the palpable nodules had been removed and a group with no nodulectomy. They concluded that “It seems likely that young, female, unencapsulated *O. volvulus* are attracted to existing nodules, settle down next to them and then become encapsulated themselves”. However, as it is admitted (i) that the female worms, once in a nodule, stay there, and (ii) that nodules form around female worms (not male worms), one may assume that the appearance of a new nodule can occur only if new females have appeared. Therefore, the lower number of new nodules recorded in the groups treated 3-monthly, when compared to the annually-treated group, results probably from an at least partial effect of ivermectin on the L4s and/or the juvenile female worms.

Specific study designs, using probably animal models, could be developed to evaluate the strength of this prophylactic effect after a single dose of IVM, which would help refine the mathematical models used to predict the impact of IVM MDA on onchocerciasis transmission intensity. Trials could also be conducted to define which treatment frequency would be required to obtain the best prophylactic effect. We found that 3-monthly treatments led to a significant reduction in the appearance of new nodules when compared to annual treatment, but the difference was not very marked. Monthly treatments would probably lead to a stronger effect, as suggested by the results of studies conducted on the *O. ochengi*/cattle model [4, 6]. Such monthly treatments have been used in studies evaluating their possible macrofilaricidal effect on *O. volvulus* [16] or their effect on *Loa loa* [17]. They probably cannot be applied on a large scale, but could be proposed to individuals visiting temporarily an onchocerciasis endemic area. Unlike loiasis which can be prevented (totally) using diethylcarbamazine (DEC) [18, 19], no drug is currently proposed to prevent onchocerciasis. Trials using DEC were conducted on chimpanzees experimentally infected with *O. volvulus* and on humans by looking at the effect of the drug on L3s, but the results were not conclusive [20].

A remaining question is why the difference of impact between 3-monthly and annual treatment is higher when the initial number of nodules is higher. Before the start of this study, some participants had more nodules than others. This variability can be explained by different levels of exposure to onchocerciasis transmission, but also by inter-individual heterogeneity in immunological response. Indeed, Tchakouté et al. [21] observed the wide variation in susceptibility between cattle (even when controlling for blackfly exposure). Some individuals are more predisposed than others to tolerate incoming parasites, with a weaker immune response allowing more L3s’ and L4s’ developing to the adult stage, and therefore leading to more nodules. During the three years of the trial, it is very unlikely that these two factors changed for the participants. Thus, it makes sense that the most infected people (i.e. with the highest initial number of nodules and highest initial MFD) at the start of the study, are also the most infected ones at the end of the trial.

Few studies tried to evaluate the impact of IVM treatment on nodules’ disappearance. Duke et al. [22] assessed this phenomenon by comparing patients who were given IVM at 150 µg/kg 3-monthly and untreated persons. They described a higher proportion of nodules that had disappeared in the treated group but the difference was not significant. Three other studies reported that nodules can disappear after repeated doses of IVM [23–25]. We did not find a difference in the number of nodules that disappeared between the treatment arms of our study, but this could be due to a lack of statistical power due to a small sample size. Further studies have to be conducted to determine the impact of repeated doses of IVM on the nodules’ disappearance.

## Conclusions

This study provides evidence that 3-monthly treatment is more effective than annual treatment to prevent the appearance of onchocercal nodules. This effect is particularly marked in individuals with a large number of nodules before treatment. Our results support, for the first time, that ivermectin has probably a prophylactic effect on the L4s and/or the juvenile female *O. volvulus* worms. When the drug is given at a three-month interval, this effect is only partial, but it might be more efficient when given at shorter intervals.

## Abbreviations

NTD: neglected tropical diseases
WHO: World Health Organization
MDA: mass drug administration
IVM: ivermectin
MFD: microfilarial density
CMFL: community microfilarial load
DEC: diethylcarbamazine
